# Emphysema Shapes a Pro-Inflammatory Immune Microenvironment in Pulmonary Adenocarcinoma: A Pilot Immune Transcriptomic Profiling Study

**DOI:** 10.3390/ijms27093958

**Published:** 2026-04-29

**Authors:** Jeong Uk Lim, Seohyeon Kim, Tai Joon An, Young Jo Sa, Hyo Rim Kim, Chan Kwon Park, Hyoung Kyu Yoon, Tae-Jung Kim

**Affiliations:** 1 Division of Pulmonary and Critical Care Medicine, Department of Internal Medicine, Yeouido St. Mary’s Hospital, College of Medicine, The Catholic University of Korea, Seoul 07345, Republic of Korea; cracovian@catholic.ac.kr (J.U.L.); shyune318@gmail.com (S.K.); archepoly@catholic.ac.kr (T.J.A.); ckpaul@catholic.ac.kr (C.K.P.); cmcyhg@catholic.ac.kr (H.K.Y.); 2 Department of Thoracic and Cardiovascular Surgery, Yeouido St. Mary’s Hospital, College of Medicine, The Catholic University of Korea, Seoul 07345, Republic of Korea; sagoon@catholic.ac.kr; 3 Department of Radiology, Yeouido St. Mary’s Hospital, College of Medicine, The Catholic University of Korea, Seoul 07345, Republic of Korea; horrim@catholic.ac.kr; 4 Department of Hospital Pathology, Yeouido St. Mary’s Hospital, College of Medicine, The Catholic University of Korea, Seoul 07345, Republic of Korea

**Keywords:** emphysema, adenocarcinoma, nanostring, tumor microenvironment, pilot study

## Abstract

Emphysema is a well-recognized risk factor for lung cancer; however, its influence on the immunologic tumor microenvironment in lung adenocarcinoma remains poorly defined. In this pilot, hypothesis-generating study, immune-related gene expression profiling was performed using archival formalin-fixed paraffin-embedded tumor specimens from 12 patients with lung adenocarcinoma, including the Never-smoker group (never-smokers without emphysema; *n* = 4), the Smoker 1 group (smokers without emphysema; *n* = 3), and the Smoker 2 group (smokers with CT-defined emphysema; *n* = 5). Expression of 770 immune-related genes was analyzed using the nCounter PanCancer IO 360 Panel (NanoString Technologies, Seattle, WA, USA). Compared with the Never-smoker group, tumors from the Smoker 1 group showed marked upregulation of SFRP1, SERPINB5, and IL6, whereas tumors from the Smoker 2 group exhibited increased expression of KIR2DL3, BLK, and WNT2B. Relative to the Smoker 1 group, the Smoker 2 group demonstrated significant upregulation of MMP7, TDO2, and CCL18. Pathway enrichment analysis revealed cytokine–cytokine receptor interaction as the most prominently enriched pathway in both smoker groups, while the IL-17 signaling pathway was preferentially enriched in the Smoker 2 group. In addition, diffusing capacity for carbon monoxide showed significant correlations with immune-related genes including IL-6 and IL-6R. Collectively, these preliminary findings suggest that lung adenocarcinoma arising in emphysematous lungs may be characterized by a distinct pro-inflammatory immune microenvironment. Given the small sample size and potential confounders, these results should be regarded as hypothesis-generating. Emphysema-associated immune remodeling may nevertheless represent an important biological factor worthy of validation in larger, independent cohorts.

## 1. Introduction

Lung cancer remains one of the leading causes of cancer-related mortality worldwide. Non-small cell lung cancer (NSCLC) accounts for approximately 70% of all lung cancer cases, with adenocarcinoma representing the most prevalent histologic subtype [1]. Despite substantial advances in molecular characterization and targeted therapies, lung adenocarcinoma exhibits marked heterogeneity in histopathology, driver mutations, and host immune responses. With the advent of immune checkpoint inhibitors (ICIs), the tumor microenvironment (TME) has emerged as a critical determinant of tumor progression and therapeutic response in lung adenocarcinoma [2]. Increasing evidence indicates that the TME is dynamically shaped by both host- and environment-related factors, including genetic background, interactions with adjacent lung tissue, and chronic inflammatory conditions [3].

NSCLC comprises multiple histologic subtypes that exhibit distinct biological and immunologic characteristics. In particular, adenocarcinoma has been shown to harbor a unique immune microenvironment with specific immune cell compositions and subtype-dependent prognostic implications [4]. These observations highlight the importance of investigating tumor–immune interactions within a single histologic subtype to avoid confounding by histology-dependent immune heterogeneity. Therefore, the present study focused exclusively on lung adenocarcinoma.

Chronic obstructive pulmonary disease (COPD) is one of the most common comorbidities in patients with NSCLC [5,6] and is consistently associated with worse clinical outcomes [7,8,9]. Epidemiologic studies indicate that patients with COPD have nearly a tenfold increased risk of developing lung cancer compared with the general population [10]. COPD is an independent risk factor for several histologic subtypes of lung cancer, and Mendelian randomization analyses suggest that COPD mediates approximately 36% of the association between smoking and lung adenocarcinoma [11]. Although emphysema-predominant COPD has been linked to a higher risk of squamous cell carcinoma and small cell lung cancer relative to non-emphysema-predominant phenotypes [12], the impact of emphysema on the immune landscape of adenocarcinoma—the most common subtype of NSCLC—remains poorly characterized.

Smoking and lung emphysema are major contributors to COPD pathogenesis [13]; however, the biological mechanisms linking COPD to lung carcinogenesis remain incompletely understood [14]. The introduction of chest computed tomography (CT) has enabled non-invasive detection and quantification of emphysema, facilitating refined phenotyping of COPD into emphysema-predominant, airflow obstruction-predominant, and mixed subtypes [15]. Wilson and colleagues demonstrated that radiographic emphysema independently increases lung cancer risk even after adjustment for airflow limitation [16]. Although reduced forced expiratory volume in one second (${\mathrm{FEV}}_{1}$) has also been associated with lung cancer risk, it remains unclear whether ${\mathrm{FEV}}_{1}$ primarily reflects emphysema severity or whether additional COPD-related processes contribute to tumor development. In contrast to COPD, which is physiologically defined by airflow obstruction, emphysema represents a structural phenotype characterized by the abnormal, irreversible enlargement of the airspaces distal to the terminal bronchioles, accompanied by destruction of the alveolar septa due to chronic inflammation rather than simple insufflation or air trapping [17,18]. Although historically diagnosed at autopsy or inferred from pulmonary function testing, modern high-resolution computed tomography (HRCT) now allows detailed in vivo imaging and automated quantification of emphysema, enabling detection of early-stage, subclinical, and specific phenotypes of the disease [18].

Genome-wide gene expression studies have provided insights into molecular alterations associated with COPD [19,20,21] and emphysema [22,23]. More recently, Tang and colleagues demonstrated that COPD can influence the immune landscape of lung cancer, particularly affecting immune cell composition within tumor and stromal compartments [24,25,26]. Emerging clinical evidence further suggests that COPD is associated with improved responses to immune checkpoint inhibitors in NSCLC, independent of smoking status [27,28]. Chronic inflammation in COPD may promote overexpression of immune checkpoint molecules on T cells within the TME, potentially increasing sensitivity to PD-1/PD-L1 blockade [28]. Nevertheless, the specific contribution of emphysema—distinct from broadly defined COPD—to immune remodeling of the TME in NSCLC remains insufficiently understood, particularly at the transcriptomic level. Furthermore, smoking itself induces immunosuppressive immune infiltration characterized by increased regulatory T cells and neutrophils [4], making it essential to distinguish the effects of smoking from those of emphysema.

Based on these observations, we hypothesized that emphysema, as a key structural and inflammatory component of COPD, may be associated with distinct immune remodeling of the tumor microenvironment in lung adenocarcinoma. Therefore, we designed the present investigation as a pilot, hypothesis-generating study using NanoString nCounter-based immune transcriptomic profiling to characterize immune-related gene expression patterns associated with emphysema in a clinically stratified cohort of lung adenocarcinoma. By comparing the Never-smoker group, the Smoker 1 group, and the Smoker 2 group, we sought to explore whether emphysema is associated with a distinct immune transcriptomic signature within the tumor microenvironment. Given the exploratory nature and small sample size of this study, the findings are intended to generate hypotheses for subsequent confirmatory investigations in larger, independent cohorts.

## 2. Results

### 2.1. Clinical Characteristics

The baseline clinical characteristics of the study cohort are summarized in Table 1. A total of 12 patients with lung adenocarcinoma were included in the analysis, comprising 4 patients in the Never-smoker group, 3 patients in the Smoker 1 group, and 5 patients in the Smoker 2 group. Seven patients (58.3%) were male, and the mean age was 63.5 years. All tumors were histologically confirmed as adenocarcinoma. Epidermal growth factor receptor (EGFR) mutations were detected in five patients (41.7%).

All patients underwent pulmonary function testing prior to surgery. No patients had stage IV disease. Based on pathologic staging, nine patients (75%) were classified as stage I, two patients (16.7%) as stage II, and one patient (8.3%) as stage III. All patients underwent surgical resection as first-line treatment, and all tumor specimens used in this study were obtained from surgical resections. One patient initially staged clinically as stage II was upstaged to stage III following postoperative pathologic evaluation.

The mean postoperative follow-up period was 39.7±15.1 months among the 11 evaluable patients, and only one patient experienced postoperative recurrence during the observation period.

### 2.2. Comparison of the Three Groups

Patients were categorized into three groups according to smoking status and the presence of emphysema on baseline chest computed tomography (CT): the Never-smoker group (never-smokers without emphysema), the Smoker 1 group (smokers without emphysema), and the Smoker 2 group (smokers with CT-defined emphysema).

All patients in the Never-smoker group were female. Among the four patients in this group, three (75%) harbored EGFR mutations, whereas EGFR mutations were detected in one of three patients (33.3%) in the Smoker 1 group and one of five patients (20%) in the Smoker 2 group. Pulmonary function parameters did not show significant differences between the groups. All patients in the Smoker 1 and Smoker 2 groups were pathologically classified as stage I, whereas the Never-smoker group included one stage III case, two stage II cases, and one stage I case (Table 2). These imbalances in sex, EGFR mutation status, and pathologic stage are further addressed in the Discussion as potential confounders.

### 2.3. Comparison of Immune-Related Gene Expression

Differential expression analysis of immune-related genes revealed distinct patterns among the study groups. Compared with the Never-smoker group, the Smoker 1 group showed 59 significantly upregulated genes and 45 downregulated genes (p<0.05). The Smoker 2 group showed 25 upregulated and 42 downregulated genes compared with the Never-smoker group. When comparing the Smoker 2 group with the Smoker 1 group, 26 genes were upregulated and 59 genes were downregulated.

**Figure 1:**
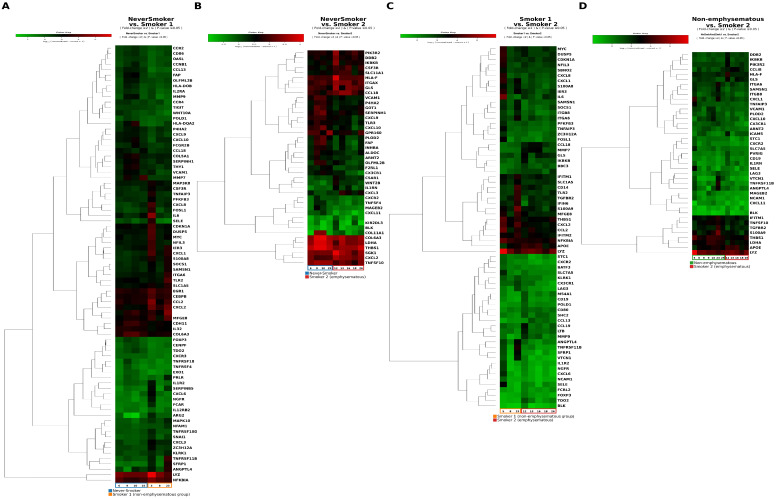
Heatmaps showing differential expression of immune-related genes across the study groups. (**A**) Never-smoker group vs. Smoker 1 group; (**B**) Never-smoker group vs. Smoker 2 group; (**C**) Smoker 1 group vs. Smoker 2 group; (**D**) Non-emphysematous group (Never-smoker and Smoker 1 groups combined) vs. Smoker 2 group. Color-coded group annotation bars above each heatmap (Never-smoker = blue, Smoker 1 = yellow, Smoker 2 = red) denote sample assignment. Each row represents a differentially expressed immune-related gene (fold-change ≥ 2, p≤ 0.05); gene names are displayed in enlarged font to ensure readability in both online and printed versions. These heatmaps illustrate that smoking and emphysema are each associated with distinct immune transcriptomic patterns, with the Smoker 2 group showing a predominantly pro-inflammatory and matrix-remodeling signature.

When the Never-smoker and Smoker 1 groups were combined as the Non-emphysematous group and compared with the Smoker 2 group, 23 genes were significantly upregulated and 49 genes were downregulated (Figure 1).

Among the genes upregulated more than two-fold compared with the Never-smoker group, SFRP1, SERPINB5, IL6, TNFRSF11B, NGFR, CXCL6, CXCL1, LYZ, CXCL8, CXCL2, and SELE were identified in the Smoker 1 group, whereas KIR2DL3 and BLK were upregulated more than two-fold in the Smoker 2 group. Compared with the Smoker 1 group, MMP7, TDO2, CCL18, BLK, MMP9, CCL13, and LTB were upregulated more than two-fold in the Smoker 2 group.

The top differentially expressed genes identified in the pairwise comparisons between the study groups are summarized in Table 3, Table 4, Table 5 and Table 6.

**Figure 2:**
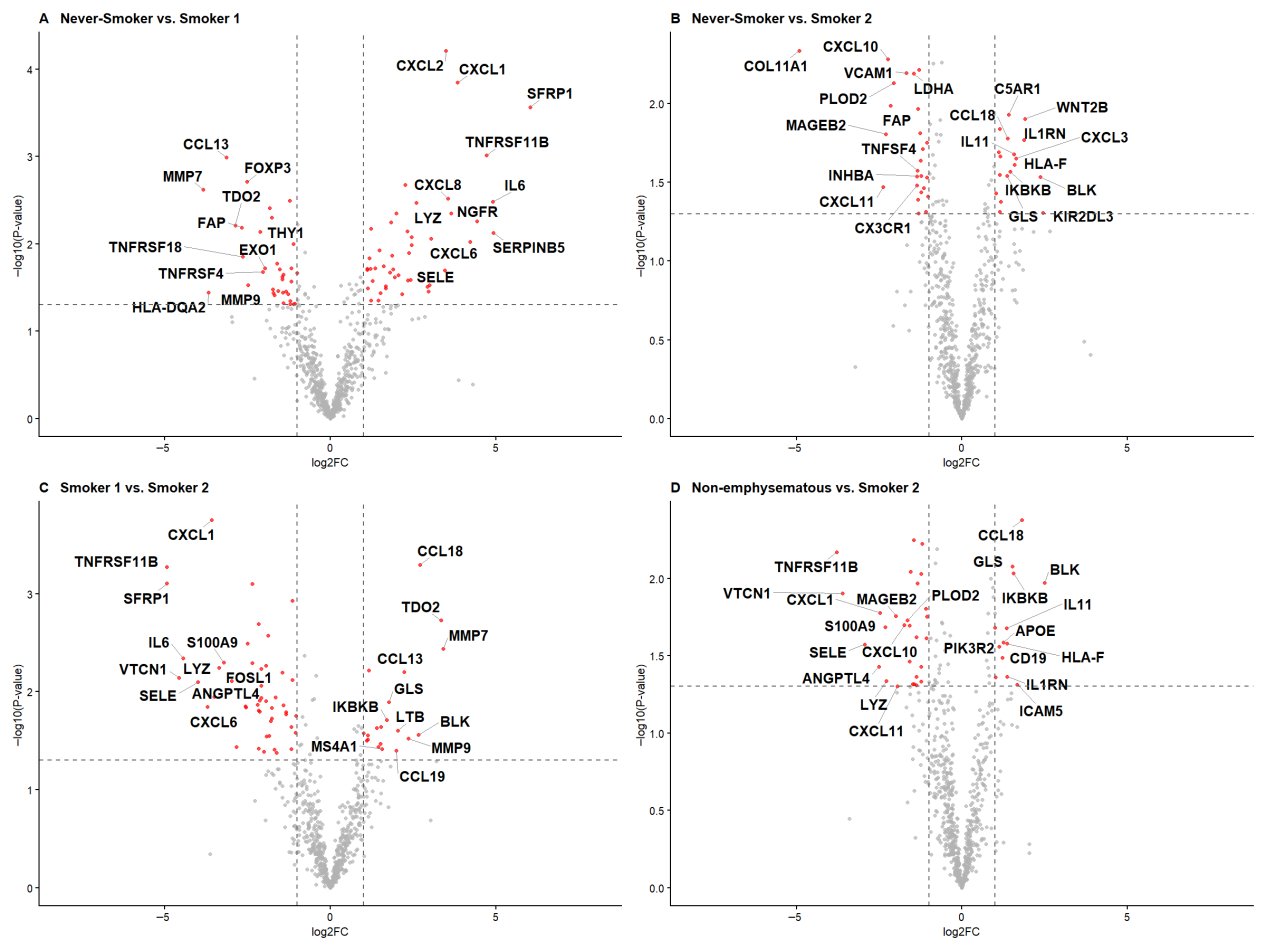
Volcano plots illustrating differential expression of immune-related genes across the study groups. (**A**) Never-smoker group vs. Smoker 1 group; (**B**) Never-smoker group vs. Smoker 2 group; (**C**) Smoker 1 group vs. Smoker 2 group; (**D**) Non-emphysematous group (Never-smoker and Smoker 1 groups combined) vs. Smoker 2 group. Dashed lines indicate significance thresholds (vertical: $|\log_{2}\mathrm{FC}|$ = 2; horizontal: *p* = 0.05). Key differentially expressed genes (SFRP1, IL6, CXCL1, CXCL8, TNFRSF11B, MMP7, TDO2, CCL18, BLK, MMP9, and VTCN1) are directly labeled in each panel. These plots highlight that the Smoker 2 group is characterized by a distinct set of pro-inflammatory and matrix-remodeling transcripts (e.g., MMP7, TDO2, CCL18, BLK) relative to non-emphysematous tumors.

When the Non-emphysematous group (Never-smoker and Smoker 1 groups combined) was compared with the Smoker 2 group, BLK was the only gene showing more than two-fold upregulation. This finding was consistent with the pairwise analyses, in which BLK was repeatedly upregulated in the Smoker 2 group. Among the genes upregulated in the Smoker 2 group, TDO2 was notable because it remained one of the top-ranked genes in the comparison with the Smoker 1 group. Volcano plots illustrating the distribution of differentially expressed genes are shown in Figure 2.

### 2.4. Pathway Enrichment Analysis

**Figure 3:**
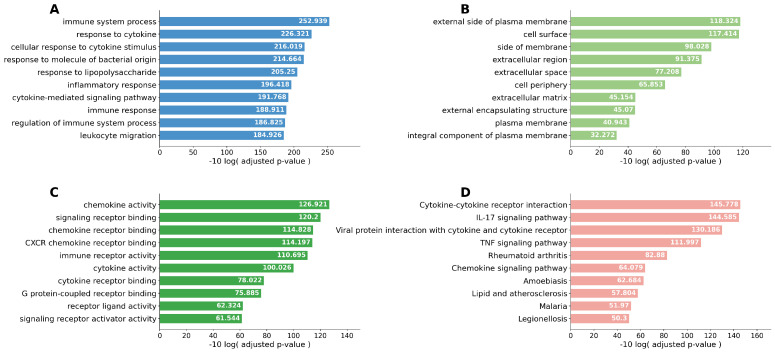
Top 10 enriched pathways identified by singular enrichment analysis for the Never-smoker group vs. Smoker 1 group comparison. (**A**) GO biological process; (**B**) GO cellular component; (**C**) GO molecular function; (**D**) KEGG pathway. Cytokine–cytokine receptor interaction emerged as the most prominently enriched KEGG pathway, consistent with smoking-related inflammatory signaling within the tumor microenvironment in the absence of emphysema.

**Figure 4:**
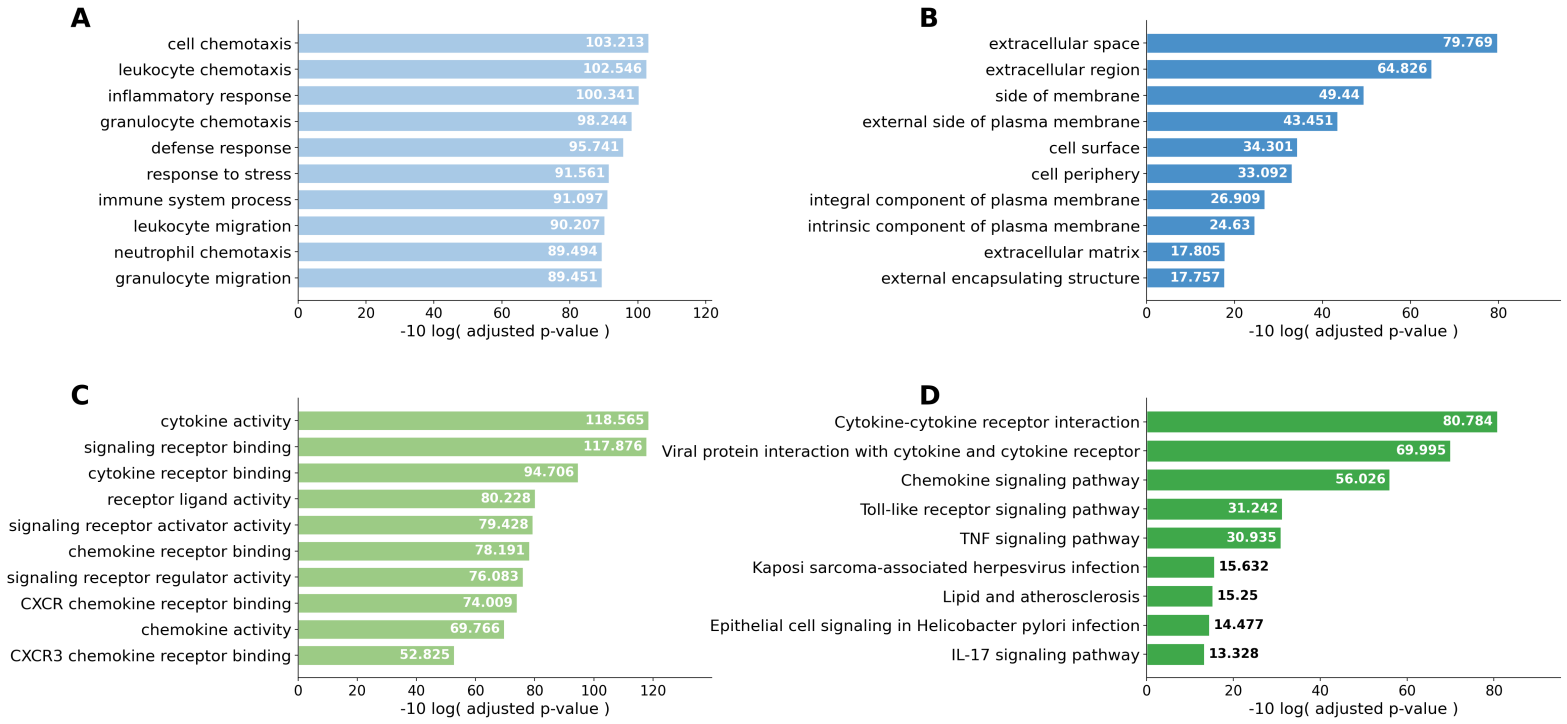
Top 10 enriched pathways identified by singular enrichment analysis for the Never-smoker group vs. Smoker 2 group comparison. (**A**) GO biological process; (**B**) GO cellular component; (**C**) GO molecular function; (**D**) KEGG pathway. Cytokine–cytokine receptor interaction and chemokine signaling pathway were prominently enriched in the Smoker 2 group, indicating robust pro-inflammatory remodeling in emphysema-associated tumors relative to never-smokers.

**Figure 5:**
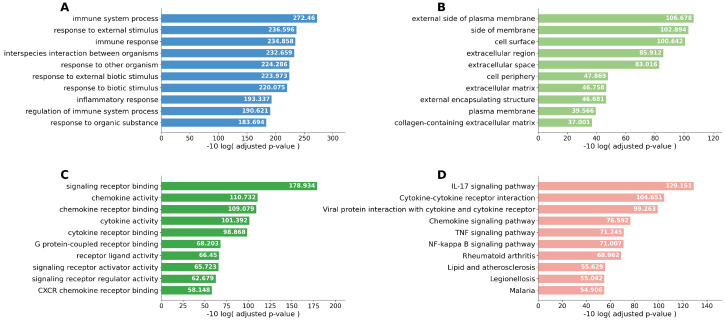
Top 10 enriched pathways identified by singular enrichment analysis for the Smoker 1 group vs. Smoker 2 group comparison. (**A**) GO biological process; (**B**) GO cellular component; (**C**) GO molecular function; (**D**) KEGG pathway. The IL-17 signaling pathway was preferentially enriched in the Smoker 2 group, supporting a distinct emphysema-associated inflammatory signature that differs from smoking alone.

**Figure 6:**
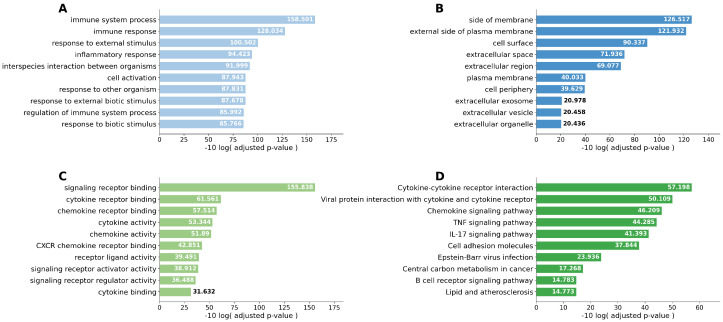
Top 10 enriched pathways identified by singular enrichment analysis for the Non-emphysematous group (Never-smoker and Smoker 1 groups combined) vs. Smoker 2 group comparison. (**A**) GO biological process; (**B**) GO cellular component; (**C**) GO molecular function; (**D**) KEGG pathway. Cytokine–cytokine receptor interaction remained the most upregulated pathway in the Smoker 2 group, further supporting emphysema-specific pro-inflammatory remodeling of the tumor microenvironment.

Singular enrichment analyses based on Kyoto Encyclopedia of Genes and Genomes (KEGG) pathways were performed. Cytokine–cytokine receptor interaction was the most frequently enriched pathway in both the Smoker 1 and Smoker 2 groups compared with the Never-smoker group. In the comparison between the Smoker 1 and Smoker 2 groups, the IL-17 signaling pathway was the most relevant pathway. When the Never-smoker and Smoker 1 groups were combined as the Non-emphysematous group and compared with the Smoker 2 group, cytokine–cytokine receptor interaction remained the most upregulated pathway. To improve readability, the pathway enrichment results are presented across four separate figures (Figure 3, Figure 4, Figure 5 and Figure 6), each corresponding to one pairwise comparison.

### 2.5. Subgroup Analyses According to PD-L1 Expression and EGFR Mutation Status

Subgroup analyses were performed to explore differences in gene expression according to PD-L1 expression and EGFR mutation status (Table 7). Five patients showed PD-L1 expression in tumor specimens, whereas seven patients did not. When gene expression was compared according to PD-L1 status, TNFRSF11B, VTCN1, SELE, CXCL6, and CXCL1 were among the top differentially expressed genes between PD-L1-positive and PD-L1-negative tumors.

In the analysis based on EGFR mutation status, five patients harbored EGFR mutations. The most upregulated genes in the EGFR wild-type group compared with the EGFR-mutant group included SFRP1, VTCN1, SERPINB5, IL6, and TNFRSF11B. Notably, several of these genes (SFRP1, IL6, SERPINB5, TNFRSF11B) were also strongly upregulated in the Smoker 1 group relative to the Never-smoker group, suggesting that part of the smoking-associated signal may be partially confounded by EGFR mutation status given the higher EGFR mutation frequency in the Never-smoker group. In contrast, genes characterizing the Smoker 2 group (BLK, TDO2, MMP7, CCL18) were not among the top differentially expressed genes in the EGFR comparison, supporting the view that these emphysema-associated transcripts may reflect emphysema-related biology relatively independently of EGFR status.

### 2.6. Correlation Between Gene Expression and Clinical Parameters

Significant correlations between clinical parameters and immune-related gene expression are summarized in Table 8. The neutrophil-to-lymphocyte ratio (NLR) showed significant correlations with NOTCH, IL6R, TP53, and IL-22RA1. C-reactive protein (CRP) levels correlated with CXCL3, IL2, IL-11, and ERBB2. Forced vital capacity (FVC, %) showed correlations with HLA-DQA1, KRAS, and CXCR4. Diffusing capacity for carbon monoxide (DLCO, %) was significantly correlated with IL-6, CXCL5, IL-6R, HLA-DQA2, SERPINH1, HLA-DPA1, PIK3CA, and IL-18.

## 3. Discussion

The present pilot study suggests that smoking history and the presence of emphysema may be associated with distinct immune-related gene expression patterns in lung adenocarcinoma. These preliminary findings indicate that both smoking exposure and emphysema-associated lung injury may influence the tumor immune microenvironment, although the small sample size precludes definitive conclusions. A key strength of this study is that all tumors consisted of surgically resected lung adenocarcinomas, enabling a focused comparison of immune-related gene expression within a single histologic subtype. Lung adenocarcinoma possesses a biologically distinct immune microenvironment compared with other NSCLC subtypes, characterized by specific immune infiltration patterns and subtype-specific prognostic implications [4]. Increasing evidence also highlights the importance of histology-specific tumor biology in NSCLC; for example, the IASLC Pathology Committee recently proposed a grading system specifically for resected invasive squamous cell carcinoma of the lung, underscoring the biological and prognostic heterogeneity among histologic subtypes of NSCLC [29]. Restricting the present analysis to adenocarcinoma therefore minimized histology-related immune heterogeneity and allowed the observed differences in immune gene expression to be attributed more directly to smoking exposure and emphysema.

COPD and lung cancer are closely interconnected diseases. Chronic inflammation associated with COPD promotes DNA damage in lung tissue and contributes to lung carcinogenesis [30,31]. Mendelian randomization analyses further suggest that COPD may act as a causal mediator rather than merely a comorbidity, accounting for approximately 36% of the association between smoking and lung adenocarcinoma, with regulatory T cells identified as a key intermediary [11]. These findings support the concept that the chronic inflammatory environment of COPD, including emphysema, may reshape the tumor immune microenvironment through alterations in immune cell populations. Within the tumor microenvironment (TME), ${\mathrm{CD}4}^{+}$ and ${\mathrm{CD}8}^{+}$ lymphocytes contribute to antitumor immunity, whereas regulatory T cells and cancer-associated fibroblasts promote immunosuppressive processes.

Previous studies have shown that COPD alters immune regulation in NSCLC. Mark et al. observed increased Th1 differentiation and elevated PD-1 expression in tumors from patients with COPD [32]. Biton et al. further reported that co-expression of PD-1 and TIM-3 on ${\mathrm{CD}8}^{+}$ T cells correlated with COPD severity and altered tumor-infiltrating lymphocyte function [33]. Although COPD did not markedly change immune cell density in the TME, it modified immune function by altering ${\mathrm{CD}8}^{+}$ tumor-infiltrating lymphocytes [33]. Similarly, McKendry et al. demonstrated increased PD-1 expression on lung ${\mathrm{CD}8}^{+}$ T cells following viral infection in COPD tissue [34], while Freeman et al. reported correlations between IL-18 receptor-positive ${\mathrm{CD}8}^{+}$ T cells and COPD severity in resected lung specimens [35]. Collectively, these studies indicate that chronic inflammatory lung disease significantly influences immune regulation within the tumor microenvironment.

Gene expression patterns observed in this study suggest that smoking and emphysema may influence the balance between immunogenic and immunosuppressive signaling in the TME. Secreted frizzled-related proteins (SFRPs) were upregulated in the Smoker 1 group compared with the Never-smoker group. Aberrant methylation of SFRP genes has been implicated in carcinogenesis, and SFRPs act as inhibitors of WNT signaling [36,37]. Interleukin-6 (IL-6), one of the most upregulated genes in the Smoker 1 group, is a pro-inflammatory cytokine that contributes to tumorigenesis through activation of the IL-6–JAK–STAT signaling pathway [38,39]. These results suggest that smoking-associated inflammation may promote tumor-supportive immune conditions even in the absence of radiologic emphysema.

In the Smoker 2 group, matrix metalloproteinase-7 (MMP-7) and matrix metalloproteinase-9 (MMP-9) were markedly upregulated compared with the Smoker 1 group. MMPs are endopeptidases involved in extracellular matrix degradation and tissue remodeling [40]. MMP-7 contributes to ECM degradation, whereas MMP-9 has been strongly associated with tumor invasion and metastasis [41,42]. Stromal MMP-9 expression has been linked to aggressive tumor behavior in lung cancer, particularly in tumors arising in emphysematous lungs [43]. Polymorphisms in the *MMP7* gene have also been associated with COPD susceptibility [44]. Macrophage-derived MMPs, including MMP-7 and MMP-9, contribute to alveolar destruction in emphysema [45]. Their upregulation in the Smoker 2 group therefore suggests that emphysema-related tissue remodeling may extend into the tumor microenvironment.

Tryptophan 2,3-dioxygenase 2 (TDO2) was also significantly upregulated in the Smoker 2 group. TDO2 catalyzes tryptophan degradation in the kynurenine pathway [46]. The resulting kynurenine suppresses ${\mathrm{CD}8}^{+}$ T cell activity and promotes PD-1 expression on effector T cells, facilitating immune evasion [47]. In NSCLC models, dual inhibition of IDO1 and TDO2 suppresses tumor growth and enhances antitumor immune responses [48]. These observations suggest that emphysema-associated inflammation may simultaneously promote extracellular matrix remodeling and immune suppression within the TME.

Another notable observation was the upregulation of B lymphocyte kinase (BLK) in the Smoker 2 group compared with both the Never-smoker and Smoker 1 groups. BLK is a Src-family tyrosine kinase involved in B cell receptor signaling and B cell development. Reduced BLK expression has been associated with advanced tumor stage and poor prognosis in lung adenocarcinoma [49]. KEGG pathway analyses indicate that BLK-related genes are enriched in TNF signaling, IL-17 signaling, and cytokine–cytokine receptor interaction pathways [49]. BLK upregulation may therefore reflect enhanced B cell activity within the TME.

B cell infiltration in lung adenocarcinoma has been linked to tertiary lymphoid structures (TLSs), which correlate with improved prognosis and enhanced responses to immunotherapy [50,51]. Within TLSs, B cells promote ${\mathrm{CD}8}^{+}$ T cell-mediated antitumor immunity through germinal center reactions and plasma cell differentiation [51]. Conversely, regulatory B cells may suppress immune responses through secretion of interleukin-10 and transforming growth factor-β [52]. These dual roles highlight the complexity of B cell-mediated immune regulation within the TME.

Pathway enrichment analyses identified cytokine–cytokine receptor interaction as the most prominent pathway in both the Smoker 1 and Smoker 2 groups compared with the Never-smoker group. Cigarette smoking is associated with systemic inflammation and elevated cytokines such as IL-8 [53]. Smoking-induced oxidative stress may further stimulate cytokine production [54,55]. Interactions between CXCL8 and its receptors CXCR1/2 promote tumor progression by enhancing cancer stem cell proliferation and self-renewal [56].

The IL-17 signaling pathway was more strongly enriched in the Smoker 2 group than in the Smoker 1 group. IL-17 cytokines contribute to host defense but also promote chronic inflammation in lung disease. Increased IL-17A-expressing cells have been reported in COPD airway epithelium [57,58]. IL-17 signaling stimulates epithelial cells and fibroblasts to produce chemokines and growth factors that can promote tumor progression depending on the surrounding microenvironment [59,60]. In a Kras-driven lung cancer model with COPD-like inflammation, IL-17C promoted neutrophil recruitment and tumor proliferation while its genetic ablation enhanced responses to anti-PD-1 therapy [61].

DLCO, a physiologic parameter reflecting emphysema severity [62,63], showed significant correlations with several immune-related genes including IL-6, IL-6R, and CXCL6. These observations suggest that structural lung destruction may be closely linked with immune signaling within the tumor microenvironment.

Although direct validation of emphysema-associated transcriptomic signatures using TCGA-LUAD or the Gene Expression Omnibus (GEO) datasets is currently not feasible—because emphysema-specific CT phenotyping is not annotated in these public cohorts—independent TCGA- and GEO-based analyses have established the tumor-biological relevance of the key emphysema-associated genes identified in our study. MMP7 is significantly upregulated at both the mRNA and protein levels in TCGA-LUAD compared with normal lung, and high MMP7 expression is associated with poor survival in multiple lung cancer GEO cohorts, including GSE5123 and GSE31210 [64]. A 159-patient NSCLC cohort further confirmed that high MMP7 expression independently predicts chemoresistance and poor survival, with more frequent overexpression in adenocarcinoma than in squamous histology [65]. Combined elevation of MMP7 and MMP9 has been associated with metastasis and reduced post-surgical survival in a 230-patient NSCLC cohort [66]. TDO2 is overexpressed in TCGA-LUAD relative to normal lung and correlates with worse overall survival, with mechanistic links to kynurenine-mediated ${\mathrm{CD}8}^{+}$ T-cell suppression [67,68]. CCL18 expression and ${\mathrm{CCL}18}^{+}$ tumor-associated macrophage infiltration are elevated in advanced lung adenocarcinoma and predict shorter survival in independent cohorts [69,70]. Loss of BLK in TCGA-LUAD correlates with poor prognosis and is enriched in TNF, IL-17, and cytokine–cytokine receptor pathways [49]—consistent with the pathway enrichment pattern observed in the present analysis. Although these external datasets do not stratify patients by emphysema status, the convergent biological patterns support the plausibility of our pilot findings and provide a framework for future confirmatory studies in emphysema-phenotyped cohorts.

Recent clinical reports suggest that COPD may be associated with improved responses to PD-1/PD-L1 immune checkpoint inhibitors in NSCLC [27,28]. Chronic inflammation in COPD may increase immune checkpoint expression on T cells, thereby enhancing sensitivity to checkpoint blockade [28]. In our cohort, the Smoker 2 group showed enrichment of pro-inflammatory pathways, increased BLK expression, and reduced expression of the co-inhibitory molecule B7-H4 (VTCN1). PD-L1 expression was detected in 3 of 5 Smoker 2 tumors compared with 2 of 4 Never-smoker tumors and 0 of 3 Smoker 1 tumors, although this difference did not reach statistical significance (p=0.229). These transcriptomic and PD-L1 observations are consistent with a potentially immune-activated phenotype; however, this remains a hypothesis that requires validation through protein-level assays (e.g., multiplex immunohistochemistry) and, ultimately, clinical outcome data from immunotherapy-treated cohorts. The present study does not provide direct evidence of differential immunotherapy responsiveness.

Several limitations should be acknowledged. First, the sample size was small (total n=12; Never-smoker group n=4, Smoker 1 group n=3, Smoker 2 group n=5) because this study was designed as a pilot investigation of immune transcriptomic differences associated with smoking and emphysema. Given the small sample size, the present analysis should be regarded as hypothesis-generating rather than confirmatory. The limited statistical power increases the probability of both type I and type II errors [71,72], and the observed transcriptomic differences require validation in larger, independent cohorts before firm biological or clinical conclusions can be drawn.

Second, the imbalance in sex and EGFR mutation status among groups represents an important confounder, as both variables are known to shape the immune microenvironment of lung adenocarcinoma. Frequency matching for pathologic stage, sex, and age was applied during selection of the Never-smoker and Smoker 1 groups from their eligible pools; however, because all five Smoker 2 cases meeting the eligibility criteria were male, complete balance for sex across all three groups was not achievable. Similarly, EGFR mutation status, which is strongly linked to sex and smoking history in East Asian lung adenocarcinoma, could not be fully balanced given the constrained pool. Notably, several genes upregulated in the Smoker 1 group (e.g., SFRP1, IL6, SERPINB5, TNFRSF11B) were also differentially expressed according to EGFR mutation status, suggesting that part of the smoking-associated signal may be partially driven by EGFR status. In contrast, genes characterizing the Smoker 2 group (BLK, TDO2, MMP7, CCL18) were not among the top differentially expressed genes in the EGFR comparison, supporting the view that these genes may reflect emphysema-related biology more robustly. Because multivariable adjustment was not feasible in the present sample size, these results must be interpreted with caution and confirmed in larger, more balanced cohorts.

Third, the pathologic stage distribution was imbalanced across groups. All Smoker 1 and Smoker 2 tumors were stage I, whereas the Never-smoker group included stage II and stage III cases. Given the small sample size, stage-stratified subgroup analysis was not feasible. However, because the stage was on average higher in the Never-smoker group, stage imbalance alone is unlikely to explain the upregulation of pro-inflammatory and matrix-remodeling genes observed in the stage I Smoker 2 tumors. This limitation nevertheless warrants confirmation in larger, stage-matched cohorts.

Fourth, emphysema was assessed by visual CT scoring rather than by quantitative densitometry. Visual scoring was performed independently by one pulmonologist and one thoracic radiologist, with disagreements resolved by consensus. Previous studies have shown that visual emphysema scoring correlates with densitometric measures (r = 0.65–0.82) [73,74], supporting its validity as a surrogate measure, although inter-reader agreement may decrease at higher emphysema extents. Quantitative densitometric analysis (e.g., percentage of low-attenuation area below −950 HU, Perc15) could not be performed because standardized thin-section reconstructions and validated quantitative CT densitometry software (such as 3D Slicer Chest Imaging Platform, https://chestimagingplatform.org, accessed on 20 April 2026) were not uniformly available across the archival imaging studies of this retrospective cohort. Quantitative densitometry offers superior reproducibility and should be incorporated in future validation studies.

Fifth, experimental validation at the protein level (e.g., immunohistochemistry, qPCR) and in emphysema-phenotyped external cohorts was not performed in this pilot study and represents an important priority for future work. Publicly available transcriptomic cohorts (TCGA-LUAD, GEO) do not currently include structured emphysema annotations, which precludes direct external validation of the emphysema-stratified signature.

Despite these limitations, this pilot study provides exploratory evidence that smoking and emphysema may be associated with distinct immune transcriptomic signatures in lung adenocarcinoma and suggests potential mechanisms by which chronic lung disease may shape tumor immunity. These hypothesis-generating findings should be interpreted within the constraints described above and tested in larger, independent cohorts with comprehensive clinical, radiologic, and molecular annotation.

## 4. Materials and Methods

### 4.1. Study Population and Tissue Selection

A total of 299 patients diagnosed with non-small cell lung cancer between 2018 and 2020 at Yeouido St. Mary’s Hospital were initially identified. Of these, 240 patients without available surgical specimens were excluded, yielding 59 patients with archival formalin-fixed paraffin-embedded (FFPE) tumor tissue from lobectomy for resectable lung adenocarcinoma. Baseline chest computed tomography (CT) imaging and smoking history were available for all cases.

Cases were screened for eligibility prior to immune transcriptomic analysis. Inclusion criteria were histologically confirmed lung adenocarcinoma, absence of neoadjuvant therapy, adequate viable tumor content on histologic review (tumor cellularity ≥ 20%), and complete clinical and radiologic data including smoking history and baseline CT imaging. Of the 59 cases reviewed, 26 were excluded for not meeting these criteria (histological subtype other than adenocarcinoma, incomplete clinical or radiologic data, absence of informed consent, or prior neoadjuvant therapy), leaving 33 eligible cases.

Smoking status was defined based on lifetime cigarette exposure. Patients who had smoked more than 100 cigarettes during their lifetime were classified as smokers, whereas those who had not were classified as never-smokers [75].

The presence of emphysema was visually evaluated on standard-dose CT scans obtained at the time of pathologic specimen acquisition by one pulmonologist and one thoracic radiologist, both blinded to clinical data, with disagreements resolved by consensus. For assessment, each lung was divided into upper, middle, and lower regions on both sides, resulting in six regions in total. Emphysema was defined as areas of low attenuation compared with the surrounding normal lung parenchyma. The extent of emphysema in each region was graded as follows: score 0 (no emphysema), score 1 (≤25%), score 2 (≤50%), score 3 (≤75%), and score 4 (>75%). The regional scores were summed to yield a total emphysema score ranging from 0 to 24. Total CT emphysema scores of 0 or 1 were considered to indicate no emphysema [76,77]. Although quantitative densitometric analysis (e.g., percentage of low-attenuation area below −950 HU) was not performed because standardized thin-section reconstructions were not uniformly available across the archival imaging series, prior studies have shown that visual scoring correlates well with densitometric quantification [73,74].

Based on smoking status and CT-defined emphysema, the 33 eligible cases were classified into three groups: the Never-smoker group (never-smokers without emphysema), the Smoker 1 group (smokers without radiologic evidence of emphysema), and the Smoker 2 group (smokers with CT-defined emphysema). From these eligible cases, a pilot cohort of 12 patients was assembled following NanoString QC filtering and optimization of RNA quality. Specifically, the final analytic cohort comprised 4 patients in the Never-smoker group, 3 patients in the Smoker 1 group, and 5 patients in the Smoker 2 group, with the resulting sample size consistent with previously reported NanoString-based pilot studies [78]. A similar group-based classification approach using NanoString profiling of non-smoker, smoker, and COPD lung tissues has been previously described [79]. Within each group, frequency matching for pathological stage, sex, and age was applied where feasible [80], and specimens with the highest RNA integrity (DV200) were preferentially selected to optimize analytical performance [81].

**Figure 7:**
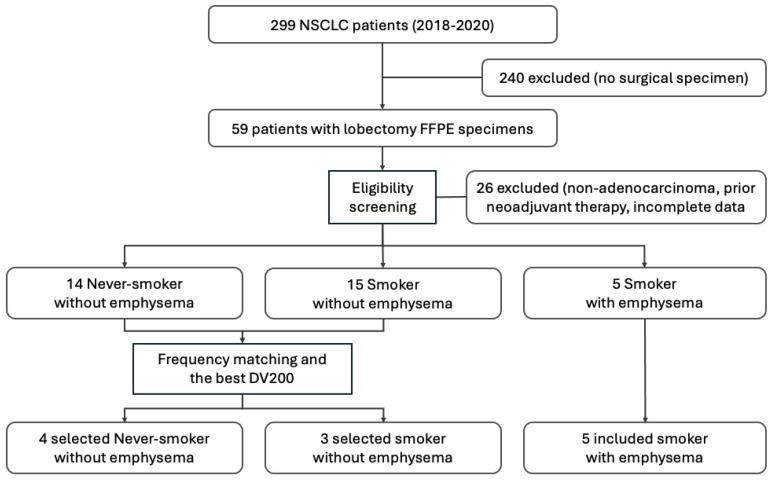
Flow diagram of patient selection.

The final analytic cohort therefore consisted of 12 patients (Never-smoker group, n=4; Smoker 1 group, n=3; Smoker 2 group, n=5) (Figure 7).

### 4.2. Tissue Processing and Quality Control for NanoString Analysis

To minimize pre-analytical variability and ensure reliable immune transcriptomic profiling from FFPE specimens, tissue- and assay-level quality control (QC) procedures were applied following established NanoString workflows and nSolver-based QC metrics [81,82]. Hematoxylin and eosin (H&E)-stained slides were reviewed by a board-certified pathologist to confirm the diagnosis, identify viable tumor areas, and verify tumor cellularity (≥20%). Macrodissection was performed from corresponding unstained FFPE sections to enrich tumor content.

Total RNA was extracted from FFPE tissue using the High Pure FFPET RNA Isolation Kit (Roche Life Science, Indianapolis, IN, USA) and quantified using a NanoDrop ND-1000 spectrophotometer (Thermo Fisher Scientific, Waltham, MA, USA). RNA integrity was assessed using DV200 values measured on an Agilent 2100 Bioanalyzer (Agilent Technologies, Santa Clara, CA, USA); samples with DV200 ≥ 30% were considered acceptable for downstream analysis. RNA input was adjusted according to DV200 values following the manufacturer’s recommendations. A total of 100–300 ng of RNA per sample was hybridized to the NanoString nCounter PanCancer IO 360 Panel (NanoString Technologies, Seattle, WA, USA) at 65 °C for 16–18 h. Cartridge preparation was performed on the nCounter Prep Station (NanoString Technologies, Seattle, WA, USA), and digital counting was carried out on the nCounter Digital Analyzer (NanoString Technologies, Seattle, WA, USA) at 555 fields of view (FOV), according to the manufacturer’s instructions.

Raw count data (Reporter Code Count [RCC] files) were evaluated using nSolver QC metrics, including imaging quality and binding density [82]. Imaging QC was assessed based on the percentage of successfully scanned FOV, while binding density was evaluated to ensure that barcode counts fell within the recommended range (0.1–2.25) for accurate quantification. In addition, positive control linearity ($R^{2}$ > 0.95) and limit-of-detection metrics based on negative controls were examined as part of the NanoString QC workflow [83]. Samples failing critical QC criteria or showing abnormal normalization factors were excluded from further analysis. After QC filtering, gene expression counts were normalized using internal positive controls and housekeeping genes prior to downstream differential expression and pathway analyses [84].

### 4.3. Data Normalization

Normalization of raw count data was performed using the nCounter Advanced Analysis module (version 2.0) within nSolver Analysis Software (version 4.0; NanoString Technologies, Seattle, WA, USA) [82,85]. First, positive control normalization was applied by computing a lane-specific scaling factor from the geometric mean of the six synthetic RNA positive control probes (ERCC spike-in controls) to correct for inter-lane technical variability in hybridization, purification, and binding efficiency. Samples with positive control normalization factors outside the acceptable range (0.3–3.0) were flagged for review.

Subsequently, codeset content normalization was performed using the panel’s built-in housekeeping genes. The most stably expressed housekeeping genes were selected using the geNorm algorithm [86], which iteratively eliminates genes with the highest expression variability to identify an optimal reference gene set. The geometric mean of the selected housekeeping genes was used to derive sample-specific normalization factors, thereby adjusting for differences in RNA input and sample quality across specimens. Background thresholding was applied based on the mean counts of negative control probes to establish the noise floor for downstream analyses. Normalized data were $\log_{2}$-transformed prior to subsequent analyses [87].

### 4.4. Differential Gene Expression Analysis

Differential gene expression analysis was conducted using the nSolver Analysis Software (version 4.0) and its Advanced Analysis module [82,85]. The Advanced Analysis module employs a simplified negative binomial regression model, which estimates fold changes and *p*-values for each gene using information from the raw count data, incorporating estimates of noise and dispersion across all samples [83]. Pairwise comparisons were performed between the three groups (Never-smoker group vs. Smoker 1 group, Never-smoker group vs. Smoker 2 group, and Smoker 1 group vs. Smoker 2 group). Adjustment for multiple testing was performed using the Benjamini–Hochberg false discovery rate (FDR) method, with an FDR-adjusted *p*-value < 0.05 considered statistically significant. Volcano plots and hierarchical clustering heatmaps were generated to visualize differentially expressed genes and expression patterns across groups.

### 4.5. Immune Cell Type Profiling

Immune cell type abundance was estimated from the normalized gene expression data using the cell type profiling module within nCounter Advanced Analysis [85]. The PanCancer IO 360 Panel includes curated gene signatures for 14 immune cell populations, including ${\mathrm{CD}8}^{+}$ T cells, ${\mathrm{CD}4}^{+}$ T cells, B cells, natural killer (NK) cells, macrophages, dendritic cells, mast cells, neutrophils, regulatory T cells (Tregs), cytotoxic cells, exhausted ${\mathrm{CD}8}^{+}$ T cells, and Th1 cells, among others [88]. Cell type abundance scores were computed as the $\log_{2}$-scaled average expression of constituent marker genes for each cell population in each sample. These scores represent relative enrichment levels rather than absolute cell counts and were used to compare immune cell composition across the three study groups.

### 4.6. Biological Pathway and Signature Scoring

Pathway activity and biological signature scores were derived using the nCounter Advanced Analysis module [85]. The PanCancer IO 360 Panel provides 48 pre-defined biological signatures encompassing key aspects of tumor–immune interactions, including the 18-gene Tumor Inflammation Signature (TIS), signatures related to interferon signaling, antigen presentation, T cell function, myeloid cell activity, immune cell adhesion and migration, stroma, proliferation, and other tumor and immune processes [89]. For each sample, signature scores were calculated as the first principal component of the constituent genes’ normalized expression values, following the manufacturer’s algorithm. The TIS was used to characterize tumors along the immune activity continuum from “cold” (immunologically quiescent) to “hot” (inflamed), and to evaluate whether Smoker 2 (emphysema-associated) tumors exhibited a distinct inflammatory profile. Pathway scores across the three groups were compared to identify differentially activated biological processes.

### 4.7. Statistical Analysis

Comparisons of continuous variables among the three groups (Never-smoker group, Smoker 1 group, and Smoker 2 group) were performed using the Kruskal–Wallis test, a non-parametric method appropriate for small sample sizes and data not assumed to follow a normal distribution. When the Kruskal–Wallis test indicated a statistically significant overall difference, post-hoc pairwise comparisons were conducted using the Dunn test with Bonferroni correction for multiple comparisons. Comparisons between two groups were performed using the Mann–Whitney *U* test. Categorical variables were compared using Fisher’s exact test. Correlations between continuous variables were assessed using Spearman’s rank correlation coefficient.

For differential gene expression, pathway scoring, and cell type profiling, *p*-values were adjusted for multiple testing using the Benjamini–Hochberg FDR method, with an adjusted *p*-value < 0.05 considered statistically significant. All statistical analyses were performed using nSolver Analysis Software (version 4.0; NanoString Technologies), its Advanced Analysis module (version 2.0), and R software (version 4.0 or later; R Foundation for Statistical Computing, Vienna, Austria). Visualizations of results, including heatmaps, volcano plots, and box plots, were generated using R packages including ggplot2 (version 3.4.4), pheatmap (version 1.0.12), and EnhancedVolcano (version 1.20.0). A two-sided *p*-value < 0.05 was considered statistically significant unless otherwise specified.

## 5. Conclusions

In this pilot, hypothesis-generating study, smoking history and emphysema were associated with distinct immune-related gene expression patterns in lung adenocarcinoma. Tumors arising in emphysematous lungs (the Smoker 2 group) showed enrichment of inflammatory and immune regulatory pathways, including cytokine–cytokine receptor interaction and IL-17 signaling, together with differential expression of genes involved in extracellular matrix remodeling (MMP7, MMP9) and immune regulation (TDO2, CCL18, BLK). These findings support the hypothesis that emphysema-associated chronic lung injury may shape the tumor immune microenvironment in lung adenocarcinoma. Given the small sample size, clinical imbalances between groups, and the absence of protein-level or emphysema-phenotyped external validation, these results should be interpreted as preliminary and hypothesis-generating rather than definitive. Larger, prospective cohorts incorporating quantitative CT densitometry, balanced clinical covariates, and experimental validation—together with integration of immunotherapy response data—are warranted to clarify the role of emphysema in modulating tumor immunity and treatment sensitivity in lung adenocarcinoma.

## Figures and Tables

**Table 1:** Baseline clinical characteristics of the study population (n=12).

Clinical Parameter	Value
Sex (male)	7 (58.3%)
Age, years	63.5 (31–79)
Pathologic subtype	
Adenocarcinoma	12 (100%)
EGFR mutation	5 (41.7%)
Pulmonary function	
${\mathrm{FEV}}_{1}$ (%)	89.4±15.9
${\mathrm{FEV}}_{1}$ (L)	2.6±0.6
FVC (%)	89.3±11.9
FVC (L)	3.4±0.9
DLCO (%)	96.5±24.9
DLCO (L)	18.6±6.1
Pathologic stage	
Stage I/II/III	9 (75%)/2 (16.7%)/1 (8.3%)
TNM staging	
T stage (T1/T2/T3/T4)	8 (66.7%)/3 (25%)/1 (8.3%)/0 (0%)
N stage (N0/N1/N2/N3)	10 (83.3%)/0 (0%)/2 (16.7%)/0 (0%)
M stage (M0/M1)	12 (100%)/0 (0%)
First-line treatment	
Surgery	12 (100%)
Adjuvant treatment	6 (50%)
Specimen type	
Surgical resection	12 (100%)
Observation time, months ^a^	39.7±15.1
Postoperative relapse ^a^	1/11 (9%)

^a^ Calculated among 11 evaluable patients. Abbreviations: EGFR, epidermal growth factor receptor; FVC, forced vital capacity; FEV_1_, forced expiratory volume in the first second; DLCO, diffusing capacity of the lungs for carbon monoxide.

**Table 2:** Comparison of clinical characteristics according to smoking status and presence of emphysema.

Clinical Parameter	Never-Smoker	Smoker 1	Smoker 2	*p*-Value
Number	4	3	5	–
Sex (male)	0 (0%)	2 (66.7%)	5 (100%)	0.010
Age (years)	72.5 (57–79)	57 (31–70)	66 (55–73)	0.235
EGFR mutation	3 (75%)	1 (33.3%)	1 (20%)	0.237
PD-L1 expression (SP263/22C3)	2 (50%)	0 (0%)	3 (60%)	0.229
Pulmonary function				
${\mathrm{FEV}}_{1}$ (L)	2.1 (2.0–2.6)	2.8 (2.4–3.1)	3.0 (1.6–3.6)	0.164
${\mathrm{FEV}}_{1}$ (%)	93.3 (75–117)	84 (75–87)	97 (57–104)	0.326
FVC (L)	2.8 (1.5–3.4)	3.6 (3.3–4.5)	4.2 (2.8–4.3)	0.071
FVC (%)	90.5 (79–110)	88 (74–97)	86 (74–105)	0.829
DLCO (%)	100.5 (69–116)	77 (77–127)	82 (73–145)	0.955
COPD	0 (0%)	2 (66.7%)	1 (20%)	0.214
Pathologic stage				
Stage I/II/III	1 (25%)/2 (50%)/1 (25%)	3 (100%)/0/0	5 (100%)/0/0	0.092
T stage (1/2/3)	2 (50%)/1 (25%)/1 (25%)	3 (100%)/0/0	3 (60%)/2 (40%)/0	0.355
N stage (0/1/2)	2 (50%)/0/2 (50%)	3 (100%)/0/0	5 (100%)/0/0	0.091
CRP	0.42 (0.35–0.49)	0.62 (0.35–1.69)	1.31 (0.21–4.76)	0.720
NLR	1.88 (1.26–15.8)	2.60 (2.02–2.86)	3.21 (0.81–24.3)	0.871

Abbreviations: EGFR, epidermal growth factor receptor; FVC, forced vital capacity; FEV_1_, forced expiratory volume in the first second; DLCO, diffusing capacity of the lungs for carbon monoxide; COPD, chronic obstructive lung disease; PD-L1, programmed death-ligand 1; CRP, C-reactive protein; NLR, neutrophil-to-lymphocyte ratio.

**Table 3:** Top differentially expressed genes between the Never-smoker group and the Smoker 1 group.

Upregulated			Downregulated		
Gene	${\mathrm{Log}}_{2}$FC	*p*-Value	Gene	${\mathrm{Log}}_{2}$FC	*p*-Value
SFRP1	6.04	0.00027	EXO1	−1.98	0.0190
SERPINB5	4.93	0.00748	TNFRSF4	−2.04	0.0209
IL6	4.91	0.0033	THY1	−2.11	0.0073
TNFRSF11B	4.72	0.000972	MMP9	−2.48	0.0296
NGFR	4.43	0.00555	FOXP3	−2.51	0.00194
CXCL6	4.22	0.00943	TNFRSF18	−2.64	0.0140
CXCL1	3.85	0.000143	FAP	−2.67	0.00651
LYZ	3.66	0.00447	TDO2	−2.87	0.00613
CXCL8	3.55	0.00302	CCL13	−3.13	0.00103
CXCL2	3.49	0.00006	HLA-DQA2	−3.67	0.0361
SELE	3.46	0.0201	MMP7	−3.83	0.00239

The Never-smoker group was used as the reference group.

**Table 4:** Top differentially expressed genes between the Never-smoker group and the Smoker 2 group.

Upregulated			Downregulated		
Gene	${\mathrm{Log}}_{2}$FC	*p*-Value	Gene	${\mathrm{Log}}_{2}$FC	*p*-Value
KIR2DL3	2.45	0.0494	TNFSF4	−1.34	0.0267
BLK	2.37	0.0293	CX3CR1	−1.36	0.0330
WNT2B	1.90	0.0125	INHBA	−1.36	0.0289
IL1RN	1.87	0.0170	LDHA	−1.46	0.0065
CXCL3	1.63	0.0223	VCAM1	−1.68	0.0064
HLA-F	1.59	0.0245	PLOD2	−2.06	0.0074
IL11	1.58	0.0210	FAP	−2.15	0.0103
IKBKB	1.46	0.0271	CXCL10	−2.24	0.0052
C5AR1	1.42	0.0118	MAGEB2	−2.30	0.0156
CCL18	1.38	0.0166	CXCL11	−2.38	0.0338
GLS	1.36	0.0287	COL11A1	−4.91	0.0046

The Never-smoker group was used as the reference group.

**Table 5:** Top differentially expressed genes between the Smoker 1 group and the Smoker 2 group.

Upregulated			Downregulated		
Gene	${\mathrm{Log}}_{2}$FC	*p*-Value	Gene	${\mathrm{Log}}_{2}$FC	*p*-Value
MMP7	3.42	0.00363	FOSL1	−2.98	0.0077
TDO2	3.35	0.00187	S100A9	−3.22	0.0050
CCL18	2.71	0.00051	LYZ	−3.36	0.00569
BLK	2.67	0.0274	ANGPTL4	−3.50	0.0113
MMP9	2.36	0.0300	CXCL1	−3.58	0.00018
CCL13	2.23	0.00632	CXCL6	−3.71	0.0143
LTB	2.04	0.0249	SELE	−3.99	0.00795
CCL19	2.00	0.0397	IL6	−4.45	0.00454
GLS	1.77	0.0127	VTCN1	−4.57	0.00716
IKBKB	1.71	0.0194	SFRP1	−4.93	0.00078
MS4A1	1.56	0.0383	TNFRSF11B	−4.93	0.00053

The Smoker 1 group was used as the reference group.

**Table 6:** Top differentially expressed genes between the Non-emphysematous group and the Smoker 2 group.

Upregulated			Downregulated		
Gene	${\mathrm{Log}}_{2}$FC	*p*-Value	Gene	${\mathrm{Log}}_{2}$FC	*p*-Value
BLK	2.50	0.0107	PLOD2	−1.64	0.0186
CCL18	1.81	0.0042	CXCL10	−1.74	0.0201
ICAM5	1.67	0.0485	CXCL11	−1.95	0.0498
IKBKB	1.56	0.00924	MAGEB2	−2.00	0.0175
GLS	1.52	0.00836	LYZ	−2.28	0.0461
IL1RN	1.37	0.0431	S100A9	−2.32	0.0207
HLA-F	1.36	0.0264	CXCL1	−2.48	0.0167
IL11	1.35	0.0210	ANGPTL4	−2.51	0.0373
APOE	1.25	0.0260	SELE	−2.93	0.0267
CD19	1.22	0.0325	VTCN1	−3.60	0.0125
PIK3R2	1.13	0.0276	TNFRSF11B	−3.78	0.00676

The Non-emphysematous group (Never-smoker and Smoker 1 groups combined) was used as the reference group.

**Table 7:** Comparison of differentially expressed genes according to PD-L1 expression and EGFR mutation status.

PD-L1 (+) vs. PD-L1 (−)			EGFR (+) vs. EGFR (−)		
Gene	${\mathrm{Log}}_{2}$FC	*p*-Value	Gene	${\mathrm{Log}}_{2}$FC	*p*-Value
Top 5 upregulated genes					
TNFRSF11B	3.58	0.0102	SFRP1	4.85	0.00179
VTCN1	3.55	0.0137	VTCN1	4.83	0.000936
SELE	3.03	0.0220	SERPINB5	4.67	0.00138
CXCL6	2.95	0.0334	IL6	4.02	0.00671
CXCL1	2.48	0.0170	TNFRSF11B	3.88	0.00545
Top 5 downregulated genes					
CST2	−1.71	0.0114	INHBA	−1.23	0.0215
TDO2	−1.71	0.0389	SERPINH1	−1.40	0.00263
CXCL5	−1.77	0.0200	BCAT1	−1.43	0.0189
IL21R	−1.79	0.0156	CXCL10	−1.77	0.0150
HLA-DQA2	−3.10	0.0152	FAP	−2.10	0.00885

Abbreviations: FC, fold change; PD-L1, programmed death-ligand 1; EGFR, epidermal growth factor receptor.

**Table 8:** Significant correlations between clinical parameters and immune-related gene expression.

Clinical Parameter	Gene	Correlation (R)	*p*-Value
NLR	NOTCH	0.748	0.005
	IL6R	0.580	0.048
	TP53	−0.804	0.002
	IL22RA1	−0.608	0.036
CRP	CXCL3	0.743	0.006
	IL2	−0.690	0.013
	IL11	−0.669	0.017
	ERBB2	−0.630	0.028
FVC (%)	HLA-DQA1	0.737	0.006
	KRAS	0.639	0.025
	CXCR4	−0.744	0.006
DLCO (%) adjusted	IL6	0.683	0.014
	CXCL6	0.666	0.018
	IL6R	0.581	0.047
	HLA-DQA2	−0.711	0.010
	SERPINH1	−0.687	0.014
	HLA-DPA1	−0.613	0.034
	PIK3CA	−0.606	0.037
	IL18	−0.585	0.046

Abbreviations: NLR, neutrophil-to-lymphocyte ratio; CRP, C-reactive protein; FVC, forced vital capacity; DLCO, diffusing capacity of the lungs for carbon monoxide.

## Data Availability

Data available on request due to restrictions.
